# Tamoxifen Activation of Cre-Recombinase Has No Persisting Effects on Adult Neurogenesis or Learning and Anxiety

**DOI:** 10.3389/fnins.2017.00027

**Published:** 2017-02-01

**Authors:** Peter Rotheneichner, Pasquale Romanelli, Lara Bieler, Sebastian Pagitsch, Pia Zaunmair, Christina Kreutzer, Richard König, Julia Marschallinger, Ludwig Aigner, Sébastien Couillard-Després

**Affiliations:** ^1^Institute of Experimental Neuroregeneration, Paracelsus Medical UniversitySalzburg, Austria; ^2^Spinal Cord Injury and Tissue Regeneration Center Salzburg, Paracelsus Medical UniversitySalzburg, Austria; ^3^Institute of Molecular Regenerative Medicine, Paracelsus Medical UniversitySalzburg, Austria; ^4^Department of Obstetrics and Gynecology, Paracelsus Medical UniversitySalzburg, Austria

**Keywords:** hippocampus, CreERT, neurogenesis, learning, memory, nestin, gender, behavior

## Abstract

Adult neurogenesis is a tightly regulated process continuously taking place in the central nervous system of most mammalian species. In neuroscience research, transgenic animals bearing the tamoxifen-inducible CreER^T2^-Lox system are widely used. In this study, we made use of a Nestin-CreER^T2^/R26R-YFP transgenic mouse model in which the CreER^T2^ activates the expression of YFP in multipotent neural stem cells upon tamoxifen application. Humoral factors, such as the levels of estrogens, have been reported to affect the hippocampal neurogenesis. The application of tamoxifen, a mixed agonist/antagonist of the estrogen receptor that permeates the blood-brain-barrier, could thus influence adult neurogenesis. Although the functions of adult neurogenesis are yet to be fully deciphered, a reciprocal interaction between rates of neurogenesis on the one hand and learning and mood regulation on the other hand, has been suggested. The impact of tamoxifen on neurogenesis and behavior was therefore addressed following five daily applications according to the open field test, the elevated plus maze, and Morris water maze. In addition, the impact of short-term tamoxifen application on progenitor cell proliferation, morphology, and fate in the neurogenic niche of the dentate gyrus were investigated. Finally, the influence of the route of administration (oral vs. intra-peritoneal) and gender-specific response were scrutinized. The sub-acute analysis did neither reveal significant differences in behavior, such as voluntary motor activity, anxiety behavior, and spatial learning, nor in cell proliferation, cell survival, dendritic arborization or maturation rate within the dentate gyrus between saline solution-, corn oil-, and tamoxifen-treated groups. Finally, neither the route of application, nor the gender of treated mice influenced the response to tamoxifen. We conclude that short tamoxifen treatments used to activate the CreER^T2^ system in transgenic mouse models does not have a measurable impact on adult neurogenesis or the here tested behavior, and is therefore appropriate for most studies in the field.

## Introduction

In rodents, as well as in humans, adult neurogenesis takes place in two neurogenic niches of the central nervous system: the subgranular zone of the dentate gyrus in the hippocampus and the subventricular zone of the lateral ventricles (Luskin, 1993; Kuhn et al., 1996). The neurogenic niches provide a supportive environment for the key events of neurogenesis, i.e., neural stem and progenitor cell proliferation, survival, and integration. Moreover, numerous factors modulated the rates of neurogenesis, e.g., growth factors, cytokines, physical activity, stress, etc. (Kempermann et al., 1997; Couillard-Despres et al., 2005; Villeda et al., 2011; Couillard-Despres, 2013; Aimone et al., 2014; Rotheneichner et al., 2014; König et al., 2016). Although the role of neurogenesis in cognition is still unclear and the source of intensive debates (Kempermann et al., 2004; Aimone et al., 2014; Yau et al., 2015) evidence suggests that neurogenesis contributes to cognitive input processing (Clelland et al., 2009), memory retention (Becker, 2005; Thuret et al., 2009), and the reliability of memory (Wiskott et al., 2006). It is noteworthy that the correlation between neurogenesis and cognition appears to be bidirectional, i.e., cognitive effort and learning stimulates the generation of new neurons as well (Gould et al., 1999).

An increasing number of transgenic tools to study adult neurogenesis are being based on the targeted expression of the Cre-recombinase fused with a mutated form of the estrogen receptor (e.g., CreER^T2^), providing a high affinity for tamoxifen, but a low affinity for endogenous estrogen (Feil et al., 1996, 1997; Indra et al., 1999). Upon binding of tamoxifen with the fusion protein, the latter translocates from the cytoplasm to the nucleus and excises genomic segments comprised between loxP sequences. An example of such transgenic systems is the nestin-CreER^T2^/R26R-YFP encoding the CreER^T2^ under the control of the nestin promoter, which is active in multipotent neural stem cells (Lagace et al., 2007). Concomitantly, this mouse model bears the yellow fluorescent protein (YFP) integrated in the ROSA26 locus for ubiquitous expression. Due to the presence of a loxP-flanked STOP cassette upstream of the YFP reporter, expression in the neural stem cells will only begin following the tamoxifen-induced excision by the recombinase (Lagace et al., 2007).

Tamoxifen is a mixed agonist/antagonist of the estrogen receptor. First synthesized in 1966 (Sneader, 2005), tamoxifen is mainly used as a therapy option for breast cancer (Cole et al., 1971; Jordan, 2003), premature puberty (Eugster et al., 1999), and female infertility (Steiner et al., 2005). Since tamoxifen can readily cross the blood-brain-barrier (BBB; Lien et al., 1991; Pareto et al., 2004), it could influence processes within the central nervous system (CNS). For instance, it was reported that patients who received 20 mg per day of tamoxifen for 5 years showed lower cognitive and memory performances compared to untreated patients (Paganini-Hill and Clark, 2000; Eberling et al., 2004; Boele et al., 2015).

Transgenic mouse models based on tamoxifen-activated recombinase systems are available since ~2 decades and were soon introduced in the field of neurology (Orban et al., 1992; Metzger et al., 1995; Tsien et al., 1996). Nevertheless, the influence of administration of the estrogen agonist/antagonist tamoxifen *per se* on adult neurogenesis has not been addressed in depth and conflicting data regarding potential influence on learning, memory, or anxiety have been reported (Chen et al., 2002a,b; Vogt et al., 2008; Zabihi et al., 2014; Azizi-Malekabadi et al., 2015). In this regard, estrogens have been previously shown to influence neurogenesis. For example in the dentate gyrus of female rats, the number of proliferating cells labeled by the application of BrdU is the highest during proestrus, e.g., at the highest estrogen levels, as compared to estrus and diestrus (Tanapat et al., 1999). On the other hand ovariectomy did not only drastically reduce the level of circulating estrogen, it also decreased the number of proliferating cells in dentate gyrus. Furthermore, administration of estrogen to ovariectomized rats improved spatial memory, a process associated with the hippocampus (Packard and Teather, 1997; Bimonte and Denenberg, 1999).

In the present report, we investigated the impact of tamoxifen administration on the open field and elevated plus maze tests, as well as the Morris water maze test. Moreover, we investigated the impact of short-term tamoxifen application on cell proliferation, cell survival, dendritic arborization, and maturation rate in the granular and the subgranular zone of the dentate gyrus, as well as the fate of the newly generated cells. In addition, the efficiency of an oral and intra-peritoneal administration was compared and potential gender-specific response was investigated.

## Materials and methods

### Animals

All experiments were performed in accordance to the guidelines of the “Directive 2010/63/EU of the European Parliament and of the Council of 22 September 2010 on the protection of animals used for scientific purposes” and were approved by the national animal care authorities.

This study was performed on 5 month-old nestin-CreER^T2^/R26R-YFP mice (Lagace et al., 2007). All mice were housed in groups of 2–5 mice of the same gender littermates per cage with *ad libitum* access to water and food under 12 h light/12 h dark cycle.

**Figure 1:**
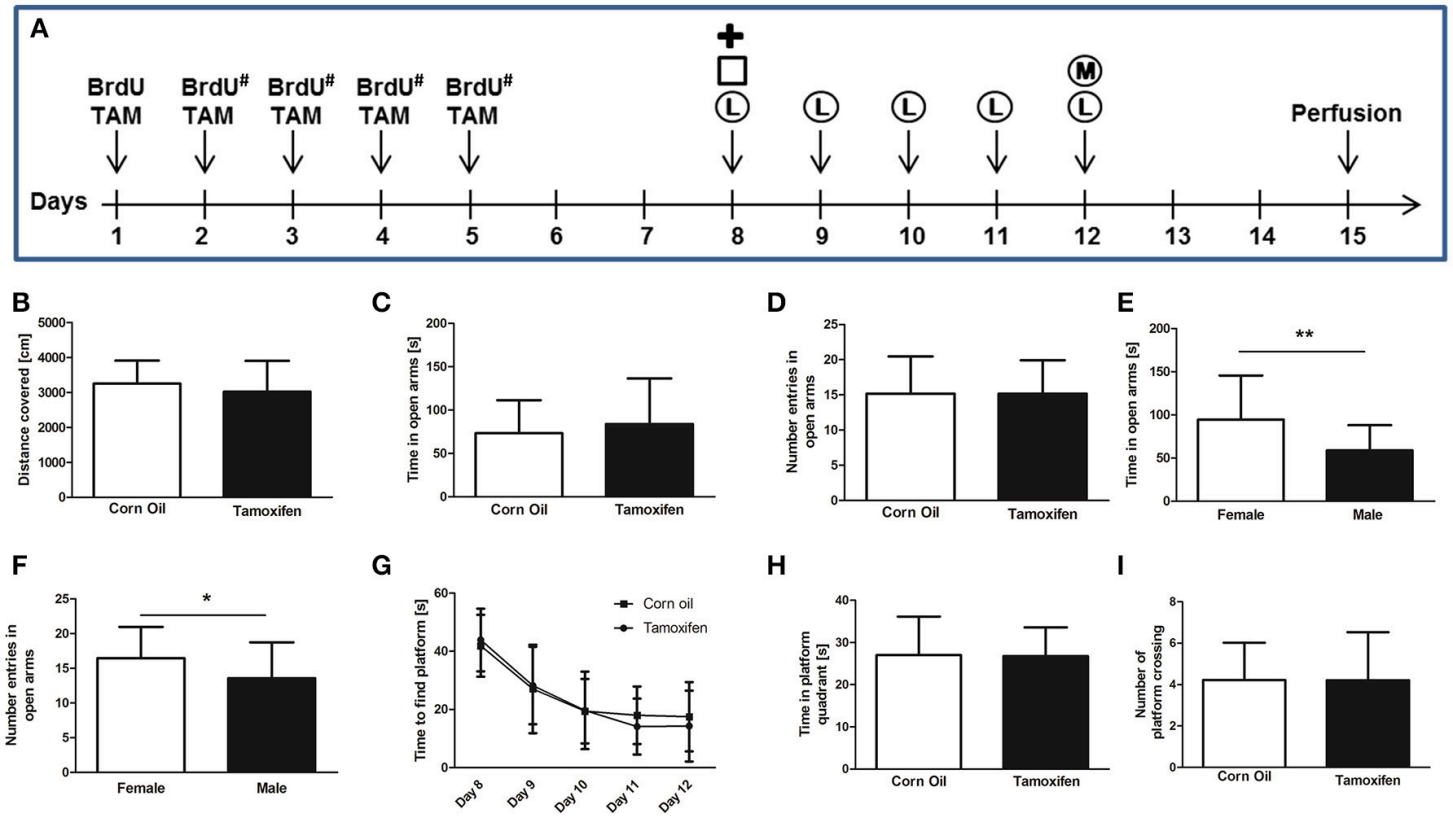
**(A)** Experimental plan of tamoxifen (TAM) and BrdU administration and behavioral tests. BrdU was injected intra-peritoneal either for 1 day, or for 5 consecutive days (#). TAM was administered intra-peritoneal or by gavage on 5 consecutive days. Behavioral tests (Plus Maze = 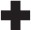, Open Field = □, Water Maze Learning = Ⓛ, and Water Maze Memory = Ⓜ) were performed from day 8 to 12. Mice were perfused on day 15. **(B)** Total distance covered in the open field test shows no significant differences in locomotion comparing corn oil and tamoxifen treatment groups. **(C**,**D)** The comparison of corn oil and tamoxifen treatment groups in the time spent in the open arm of the elevated plus maze and the number of entries in the open arms reveals no significant differences in levels of anxiety. The same result was observed by comparing the saline solution-treated group with either corn oil-treated or tamoxifen-treated mice (data not shown). **(E,F)** Gender-specific analysis of elevated plus maze data revealed significant differences in anxiety levels of females compared to males according to the time spent in open arms (*p* = 0.0065^**^) and number of open arm entries (*p* = 0.0465^*^). **(G–I)** Learning and memory performances were analyzed using the Morris water maze test. **(G)** Learning curve (time to find the platform) from day 8 to 12 revealed no learning differences between corn oil and tamoxifen treated mice, as analyzed by two-way ANOVA. Following removal of the platform on day 12, no differences in memory according **(H)** to the time spent in platform quadrant and **(I)** number of platform crossings were noticed between the tamoxifen and corn oil treated mice, as well as the saline-treated animals (data not shown).

Experiment 1: Tamoxifen (100 mg/kg bodyweight; 10 mg/ml stock solution; Sigma-Aldrich) dissolved in corn oil (Sigma-Aldrich), or corn oil as vehicle control, was administered daily from day 1 to 5 by intra-peritoneal injection or stomach gavage. Experiment 2: To investigate the possible effect of corn oil *per se* on proliferation and survival of newborn neurons, a second experiment was performed on 5 month-old C57BL6 mice which received five consecutive daily intra-peritoneal injections of either tamoxifen (100 mg/kg bodyweight), corn oil or saline solution (0.9% NaCl). In addition, 5-bromo-2′-deoxyuridine (BrdU; 50 mg/kg bodyweight; 10 mg/ml stock solution; Sigma-Aldrich) was injected intra-peritoneal either on 1 single day (Exp. 1), or daily from day 1 to 5 (Exp. 2) to label proliferating cells (Figure 1A).

### Histology

On day 15, mice were transcardially perfused with 0.9% NaCl for 5 min followed by 0.1 M phosphate buffered 4% paraformaldehyde pH 7.4 for 10 min. Brains were dissected and post-fixed in the same solution overnight at 4°C and then transferred in 0.1 M phosphate buffered 30% sucrose solution pH 7.4 at 4°C for at least 48 h. Brains were cut in 40 μm sagittal sections using a sliding microtome (Leica) on dry ice and sections were stored at −20°C in cryoprotectant (25% v/v glycerol, 0.05 M sodium phosphate buffer pH 7.4, 25% v/v ethylene glycol) until processing.

Immunohistological analyses were performed as previously described (Couillard-Despres et al., 2005) using the following antibodies and kits: chicken anti-GFP (Invitrogen, 1:500), rabbit anti-DCX (Cell Signaling Technology, 1:300), mouse anti-NeuN (Millipore, 1:500), rat anti-BrdU (AbD Serotec, 1:500), mouse anti-PCNA (Santa Cruz Biotechnology, 1:500), donkey anti-chicken Alexa 488 (Jackson Immuno Research, 1:1000), donkey anti-rabbit Alexa 568 (Invitrogen, 1:1000), donkey anti-rat Rhodamin Red (Jackson Immuno Research, 1:1000), donkey anti-rat Cy5 (Jackson Immuno Research, 1:1000), donkey anti-mouse Alexa 647 (Invitrogen, 1:1000), rabbit anti-rat biotinylated (Vector Laboratories, 1:1000), donkey anti-mouse biotinylated (Jackson Immuno Research, 1:1000), goat anti-rabbit biotinylated (Vector Laboratories, 1:1000), VECTASTAIN ABC System and DAB Peroxidase Substrate kit (Vector Laboratories) and 4′6-diamidino-2-phenylindole (DAPI, Sigma-Aldrich, 0.5 μg/ml).

The total number of labeled cells in the granular and subgranular layer of the dorsal dentate gyrus per hemisphere was extrapolated using stereological techniques on every fifth or every tenth section (Couillard-Despres et al., 2005). Bright-field micrographs were acquired with an IX81 microscope (Olympus) using the Volocity Software (Perkin Elmer). Fluorescent micrographs were obtained and analyzed with a LSM 700 confocal microscope and ZEN 2011 Black Software (Carl Zeiss) and ImageJ Software 1.46r (National Institutes of Health, USA).

For morphological analysis immature neurons were identified by their expression of doublecortin (DCX^+^). Bright-field micrographs were acquired with a Nikon Eclipse E600 microscope (objective: 100 × /1.3 oil). For each investigated DCX^+^ cell a minimum of three images of different focal planes was taken and merged using Helicon Focus 6 software. Individual neurites were traced using ImageJ (Schneider et al., 2012) in combination with the NeuronJ plugin (Meijering et al., 2004). Primary neurite length was determined and groups were compared using one-way ANOVA. For each group a minimum of six mice was taken to perform morphological analysis of DCX^+^ cells.

### Behavioral and cognitive tests

#### Open field

The open field test for spontaneous motor activity was performed on day 8 and analyzed using a tracking software (EthoVision 2.3.19, Noldus). Mice were given 5 min for free exploration on a circular arena of 100 cm diameter. Arena was cleaned between each trial to avoid olfactory cues.

#### Elevated plus maze

The elevated plus maze test was performed on day 8 to evaluate the anxiety level of mice. The maze was set 50 cm above floor level and presented four arms (50 × 10 cm), two opened and two closed arms with walls of 40 cm height. Maze was cleaned between each trial. Mice were given 5 min of free exploration starting in the central intersection facing an open arm. Tracking software (EthoVision 2.3.19, Noldus) was used to record and analyze behavior of the mice in the maze during the trial.

#### Morris water maze

The Morris water maze test was used to assess spatial learning and memory. The maze consisted in a 108 cm round basin filled with 20 cm deep water (21 ± 1°C) made opaque with milk powder. A 10 × 10 cm square platform located 1 cm below the surface was positioned in the maze. Big black cue symbols were put on the each wall of the testing room for orientation and the experimental setup was not changed throughout the learning session. Water maze trials (maximum 60 s) for learning were performed twice a day for a total of 10 trials from day 8 to 12 after first tamoxifen administration using one of four starting quadrant selected randomly. Before starting the learning experiment each mouse was put in the water and given 60 s to swim and locate the submerged platform. If the platform was not detected within 60 s the animal was guided onto the platform and allowed to remain there for 10 s. On day 12, an additional memory paradigm was performed by removing the platform and mice were tracked for 60 s. The swim path and velocity was recorded (EthoVision 2.3.19, Noldus) for every session. In the absence of the platform during the last session, the time spent in the platform quadrant and the “frequency of crossing” at the exact former position of the platform was additionally calculated.

### Statistics

Statistical analyses were performed using two-tailed unpaired *T*-test with a 95% confidence interval (GraphPad PRISM 5.0, GraphPad Software Inc.). The learning curves of the Morris water maze test, the comparison of the number of BrdU^+^ cells in relation to the duration of BrdU^+^ administration were analyzed using a two-tailed one-way or two-way ANOVA with Bonferroni *post-hoc* test. For the analysis of the proportions of BrdU^+^/DCX^+^ and BrdU^+^/NeuN^+^, as well as BrdU^+^/DCX^+^/NeuN^+^ cells between the different groups a Chi^2^-test was used. Graphs show mean values with standard deviation as error bars. Significance was *p* > 0.05 ns, *p* < 0.05^*^, *p* < 0.01^**^, and *p* < 0.001^***^.

## Results

### Impact of short tamoxifen treatments on motor activity, anxiety, and spatial learning

Spontaneous motor activity was evaluated in the open field test according to the total distance covered during a 5 min trial on day 8. Mice treated with tamoxifen for 5 consecutive days did not show significant difference in the total distance covered compared to corn oil treated groups (3251 ± 662 cm corn oil (*n* = 23) vs. 3020 ± 881 cm tamoxifen (*n* = 24), *p* = 0.32; Figure 1B), or the saline solution treated groups (data not shown). Similarly, no significant differences associated with gender or route of administration were detected within or between the treatment groups (data now shown).

The elevated plus maze test is a well-established method to evaluate the anxiety level according to the preference for open or closed arms (Pellow et al., 1985). Analyses performed on day 8 revealed no significant differences between the corn oil- or tamoxifen-treated groups in regard to time spent in open arms [73 ± 38 s corn oil (*n* = 23) vs. 84 ± 52 s tamoxifen (*n* = 24), *p* = 0.43; Figure 1C], in the number of open arm entries [15 ± 5 corn oil (*n* = 23) vs. 15 ± 5 tamoxifen (*n* = 24) *p* = 1.00; Figure 1D], nor in the total distance covered [1781 ± 297 cm corn oil (*n* = 23) vs. 1707 ± 335 cm tamoxifen (*n* = 24), *p* = 0.42]. Similarly, no significant differences were found between saline solution-treated groups and tamoxifen-treated groups (data not shown). However, females spent more time in open arms and entered open arms more frequently compared to males irrespective of treatment and route of application [time spent in open arms 95 ± 51 s females (*n* = 26) vs. 59 ± 29 s males (*n* = 21), *p* = 0.0065^**^ Figure 1E; number of open arm entries 16 ± 5 females (*n* = 26) vs. 14 ± 5 males (*n* = 21), *p* = 0.0465^*^ Figure 1F].

Morris water maze test (two trials daily) was performed from days 8 to 12 to assess spatial learning. The time required to find the hidden platform decreased from day 8 to 12 in both corn oil- and tamoxifen-treated groups (corn oil *n* = 23; tamoxifen *n* = 24; *p* < 0.0001^***^; Figure 1G). No significant difference of time-to-platform could be detected between the treatment groups (*p* = 0.64). No significant differences associated with gender or administration route were observed for these parameters within or between the treatment groups (data now shown).

On day 12, spatial memory was further assessed during a 60 s trial by measuring the total time spent in the platform quadrant, as well as frequency of crossing the platform area, following removal of the platform from the basin. No significant differences in the time spent inside the platform quadrant [27 ± 9 s corn oil (*n* = 23); 27 ± 7 s tamoxifen (*n* = 24), *p* = 0.93; Figure 1H], or in the number of crossing of the platform area [4 ± 2 corn oil (*n* = 23) and 4 ± 2 tamoxifen (*n* = 24), *p* = 0.99; Figure 1I] could be detected between the corn oil- and tamoxifen-treated groups. Similarly, no significant difference could be detected between saline-solution treated groups and corn oil-treated groups, as well as between saline solution-treated groups and tamoxifen-treated groups (data not shown). No significant differences associated with gender or administration route were observed for these parameters within or between the treatment groups (data not shown).

### Impact of tamoxifen on hippocampal neurogenesis

**Figure 2:**
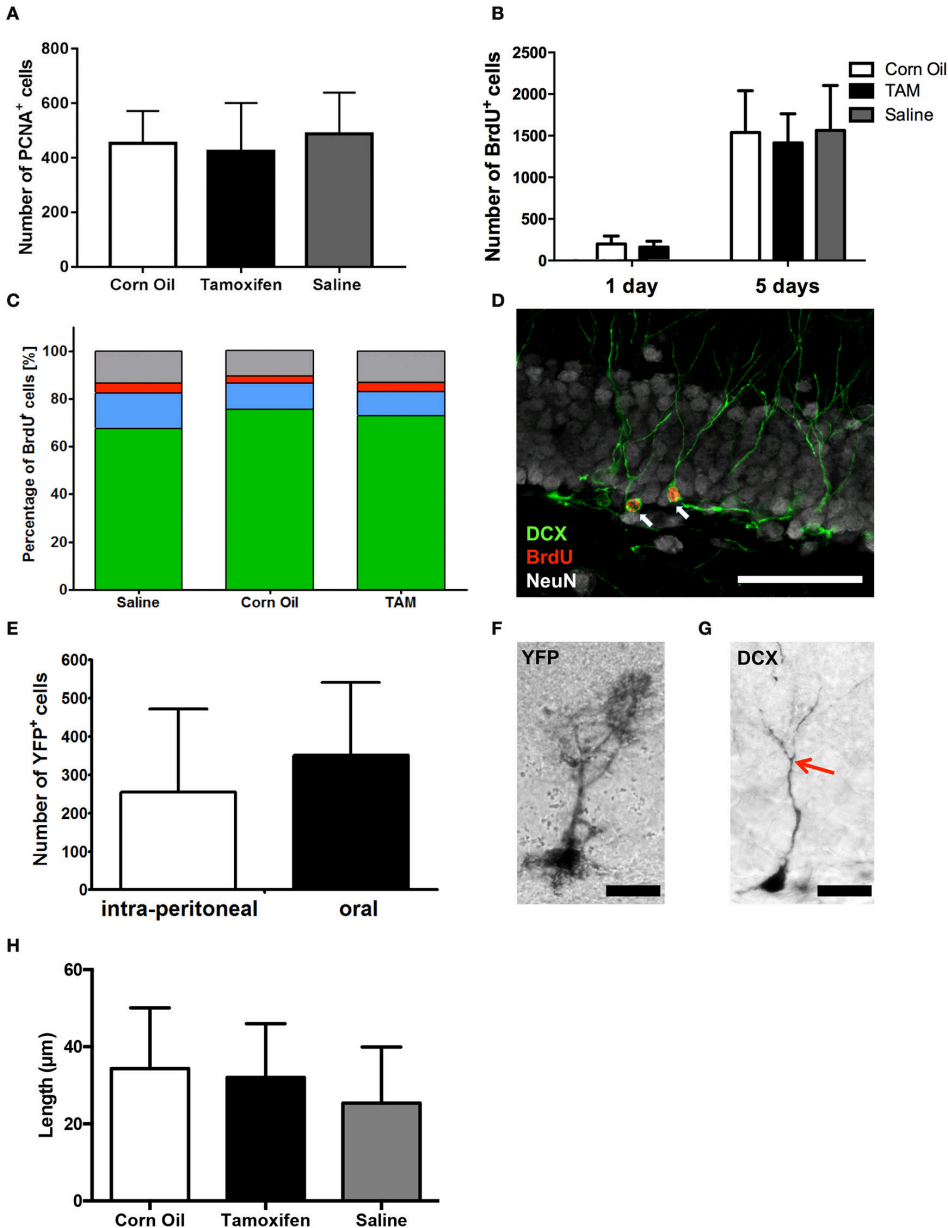
**Immunohistological analysis of (A)** cell proliferation (PNCA^+^) and **(B)** survival (BrdU^+^) in the dorsal dentate gyrus revealed no differences between saline, corn oil and tamoxifen treated mice after single BrdU administration or a BrdU administration for 5 consecutive days. **(C)** Chi^2^-Test showed independency of cell fate of BrdU^+^ cells between saline, corn oil, and tamoxifen treated mice comparing the number of cells expressing DCX and/or NeuN on day 15. **(D)** Representative immunohistological staining of the dentate gyrus showing BrdU-labeled (red) immature neurons expressing DCX (green) and the surrounding mature neurons detected with anti-NeuN (white; scale bar = 50 μm). **(E)** Analysis of the number of cells expressing YFP in the dorsal dentate gyrus revealed no significant differences in the efficacy of CreER^T2^ activation via intra-peritoneal administration of tamoxifen compared to gavage. **(F)** Representative detection of YFP using a DAB staining in the granular layer of the dentate gyrus showing YFP^+^ radial glia-like cells (scale bar = 20 μm). **(G)** DAB staining in the granular layer of the dentate gyrus identified DCX^+^ immature neurons enabling individual tracing of primary neurites (end of primary neurite marked with red arrow; scale bar = 20 μm). **(H)** Analysis of the primary neurite length of DCX^+^ immature neurons within the dentate gyrus revealed no significant morphological difference between saline solution-, corn oil-, and tamoxifen-treated mice.

The number of cells proliferating at the time of perfusion (day 15) was estimated based on the expression of the proliferation marker PCNA. The number of PCNA+ cells detected in the dorsal dentate gyrus did not significantly differ between corn oil-, saline- and tamoxifen-treated mice [487.5 ± 151.6 cells saline (*n* = 8); 453.3 ± 118.1 cells corn oil (*n* = 9); 423.8 ± 177.5 cells tamoxifen (*n* = 8), *p* = 0.6995; Figure 2A]. No significant differences associated with gender or administration route were observed in the number of PCNA-labeled cells within or between the treatment groups (data now shown).

We further scrutinized the survival of newly generated cells labeled with BrdU either administered on day 1, or for 5 consecutive days, still remaining in the dorsal dentate gyrus after 2 weeks. Corn oil-treated mice retained 199 ± 96 BrdU^+^ cells, whereas 162 ± 69 cells were detected in the tamoxifen-treated mice after 1 day BrdU administration (corn oil *n* = 15; tamoxifen *n* = 17; *p* = 0.23; Figure 2B). Moreover, BrdU administration for 5 consecutive days did not lead to any significant differences in BrdU^+^ cell numbers between saline solution-, corn oil-, and tamoxifen-treated mice [saline solution: 1563 ± 180 BrdU^+^ cells (*n* = 9); corn oil: 1538 ± 502 BrdU^+^ cells (*n* = 10); tamoxifen: 1413 ± 351 BrdU^+^ cells (*n* = 10); *p* = 0.75; Figure 2B]. Similarly, no significant differences associated with gender or administration route were observed in the number of surviving BrdU-labeled cells within or between the treatment groups (data now shown).

Morphology of DCX^+^ cells was analyzed by semi-automatic individual neurite tracing (Meijering et al., 2004) and compared between all three groups using one-way ANOVA. The primary neurite length of DCX^+^ cells in the dorsal dentate gyrus did not differ significantly between saline solution-, corn oil-, and tamoxifen-treated mice [saline solution: 25.4 ± 14.5 μm (*n* = 22); corn oil: 34.3 ± 15.8 μm (*n* = 20); tamoxifen: 32.0 ± 14.0 μm (*n* = 19); one-way ANOVA of the groups *p* = 0.14; Figures 2G,H].

In addition, we scrutinized the fate of the newly generated cells labeled with BrdU for 5 consecutive days. Neither the percentage of cell adopting a neuronal fate, as determined by the expression of DCX, nor the rate of neuronal maturation visualized by the induction of NeuN expression were affected by tamoxifen treatment compared to the saline as well as corn oil-treated group [saline: 493 DCX^+^ cells, 108 DCX^+^/NeuN^+^ cells, 30 NeuN^+^ cells (total *n* = 729 cells), corn oil: 502 DCX^+^ cells, 73 DCX^+^/NeuN^+^ cells, 20 NeuN^+^ cells (total *n* = 665 cells); tamoxifen: 441 DCX^+^ cells, 60 DCX^+^/NeuN^+^ cells, 24 NeuN^+^ cells (total *n* = 604 cells); Chi^2^-test *p* = 0.07; Figures 2C,D]. There were no significant differences associated with gender or route administration in the fate of BrdU^+^ cell, either within or between the treatment groups (data now shown).

### Impact of tamoxifen's route of administration

Although no differences in voluntary motor activity, anxiety behavior, spatial learning, and neurogenesis have been detected between mice receiving intra-peritoneal or gavage tamoxifen applications on 5 consecutive days, it remained to be determined if both routes of administration activate the CreER^T2^ system to the same extent. Two weeks following five daily applications of tamoxifen, we observed 255 ± 216 cells expressing YFP in the dorsal dentate gyrus of mice injected intra-peritoneal (*n* = 4). Administration by gavage resulted in an equivalent number of labeled cells (351 ± 189, *n* = 9, *p* = 0.43; Figures 2E,F). Gender-specific analysis did not show significant differences of recombination efficacy (data not shown).

## Discussion

Transgenic animal models are widely used in the field of neurosciences and tamoxifen-inducible CreER^T2^-Lox systems constitute an innovative and elegant method to activate or deactivate genes at a specific time point. However, in order to correctly interpret the function of the targeted gene, it is crucial to determine potential impacts of tamoxifen *per se* on behavior and neural processes. In this report, we used a Nestin-CreER^T2^/R26R-YFP mouse model (Lagace et al., 2007) to address the sub-acute impact of short-term tamoxifen administration on the adult hippocampal neurogenesis and behavior, i.e., open field, elevated plus maze, and Morris water maze tests. The latter three address voluntary motor activity, anxiety behavior, and spatial learning, respectively.

In this study, mice were perfused and analyzed 2 weeks after five daily applications of tamoxifen or vehicles, such as corn oil or saline solution. At this time point, no difference between the treatment groups in the number of cells proliferating (PCNA^+^) could be detected in the subgranular zone of the dorsal dentate gyrus where neural progenitors reside. Similarly, within the dentate gyrus, survival of newly generated cells labeled with BrdU for either one or 5 consecutive days did not differ between the two treatment groups. Furthermore, fate and maturation rate of the newly generated cells appeared similar in the three treatment groups arguing against a significant lasting influence of tamoxifen on adult hippocampal neurogenesis. Moreover, no significant differences in the morphology of the immature hippocampal neurons could be detected between saline solution-treated mice and neither the corn oil nor the tamoxifen groups. Despite our histological observation in the dentate gyrus, tamoxifen administration could provoke neurogenesis-independent modification of behavior and spatial learning. Consistent with a previous report (Vogt et al., 2008), none of the parameters investigated using our behavioral test battery indicated differences between the tamoxifen and the two vehicle-treated groups. This observation supports the hypothesis that short-term tamoxifen application, as well as short-term corn oil application does not influence behaviors when assessed at a sub-acute time point and therefore will not interfere with most experimental readouts.

Taken that tamoxifen interacts with the estrogen receptor, we further scrutinized for putative gender-specific differences in our readout parameters. Our analysis substantiated an earlier report showing that females were less anxious in the elevated plus maze test, as compared to males (Võikar et al., 2001). This difference, however, was independent of the treatment received. Apart from this gender-associated difference in anxiety, no further differences between males and females could be recognized in the other behavioral and histological parameters investigated.

Finally, we compared two alternative routes of tamoxifen administration, i.e., the intra-peritoneal injection and oral gavage. Detailed analyses did not reveal significant differences in behaviors or adult neurogenesis associated with the route of application, given the sub-acute time point analyzed here. Furthermore, we compared the respective efficacy of CreER^T2^ activation based on the number of cells expressing the reporter gene YFP in the dentate gyrus and we could not detect a significant difference associated with the route of tamoxifen application.

In conclusion, within the time-frame investigated, no differences in adult hippocampal neurogenesis or spontaneous motor activity, anxiety, and spatial learning could be identified in mice that previously received tamoxifen on 5 consecutive days, as compared to vehicle-treated mice. The equivalent efficiency in CreER^T2^ activation following intra-peritoneal and oral administration of tamoxifen argues for an oral application as first choice, since the corn oil used as vehicle for tamoxifen accumulates in the abdominal cavity for at least 2 weeks following intra-peritoneal application which could lead to local inflammation. In summary, we conclude that the short-term application of tamoxifen does not significantly interfere with adult neurogenesis or anxiety and spatial learning when assessed at the sub-acute time point tested here. This allows for the use of available CreER^T2^-Lox systems in their full potential for the investigation of adult neurogenesis.

## Author contributions

SC, LA, and JM designed the study. PeR, PaR, SP, LB, PZ, and CK performed behavioral and histological analyses. PeR, SP, LB, RK, and SC interpreted the data. PeR and SC wrote the manuscript and PaR and RK revised the manuscript.

## Funding

This study was made possible through the generous funding of the Propter Homines Foundation (Liechtenstein), the State Government of Salzburg (Austria), through funding from the European Union's Seventh Framework Program under grant agreements No. HEALTH-F2-2011-279288 (IDEA), No. FP7-REGPOT-316120 (GlowBrain), the Austrian Science Fund FWF Special Research Program (SFB) F44 (F4413-B23) “Cell Signaling in Chronic CNS Disorders,” the FWF Hertha-Firnberg Postdoctoral programme No. T736-B24, and the research fund from the Paracelsus Medical University PMU-FFF (R-14/04/063-KÖN; R-14/03/060-BIE; E-15/21/109-COU).

### Conflict of interest statement

The authors declare that the research was conducted in the absence of any commercial or financial relationships that could be construed as a potential conflict of interest.
